# Determinants of home delivery in Nepal – A disaggregated analysis of marginalised and non-marginalised women from the 2016 Nepal Demographic and Health Survey

**DOI:** 10.1371/journal.pone.0228440

**Published:** 2020-01-30

**Authors:** Bikash Devkota, Jasmine Maskey, Achyut Raj Pandey, Deepak Karki, Peter Godwin, Pragya Gartoulla, Suresh Mehata, Krishna Kumar Aryal

**Affiliations:** 1 Policy Planning and Monitoring Division, Ministry of Health and Population, Kathmandu, Nepal; 2 DFID Nepal Health Sector Programme 3/Monitoring Evaluation and Operational Research, Abt Associates, Kathmandu, Nepal; 3 UK Department for International Development Nepal, Kathmandu, Nepal; Golden Community, NEPAL

## Abstract

**Introduction:**

In Nepal, a substantial proportion of women still deliver their child at home. Disparities have been observed in utilisation of institutional delivery and skilled birth attendant services. We performed a disaggregated analysis among marginalised and non-marginalised women to identify if different factors are associated with home delivery among these groups.

**Materials and methods:**

This study used data from the 2016 Nepal Demographic and Health Survey. It involves the analysis of 3,837 women who had experienced at least one live birth in the five years preceding the survey. Women were categorised as marginalised and non-marginalised based on ethnic group. Bivariate and multivariable logistic regression analysis were performed to identify factors associated with home delivery.

**Results:**

A higher proportion of marginalised women delivered at home (47%) than non-marginalised women (26%). Compared to non-marginalised women (35%), a larger proportion of marginalised women (64%) felt that it was not necessary to give birth at health facility. The multivariable analysis indicated an independent association of having no or basic education, belonging to middle, poorer and the poorest wealth quintile, residing in Province 2 and not having completed of four antenatal care visits per protocol with home delivery among both marginalised and non-marginalised women. Whereas residing in a rural area, residing in Province 7, and at a distance of >30 minutes to a health facility were factors independently associated with home delivery only among marginalised women.

**Conclusion:**

We conclude that poor education, poor economic status, non-completion of four ANC visits and belonging to Province 2 particularly determined either group of women to deliver at home, whereas residing in rural areas, living far from health facility, and belonging to Province 7 determined marginalised women to deliver at home. Preventing mothers from delivering at home would thus require focusing on specific geographical areas besides considering wider socio-economic determinants.

## Introduction

Globally, approximately 830 women die every day during pregnancy and child birth due to preventable causes. Around 99% of maternal deaths occur in developing countries with South Asia attributing almost one third of total deaths. Life time risk of maternal deaths is 1 per 4,900 in developed countries while it is 1 per 180 in developing countries [1].

Between 2011 and 2016, both the proportion of institutional delivery and deliveries attended by skilled birth attendant (SBA) rose by approximately 22%. In the same period, the maternal mortality ratio (MMR) decreased from 539 to 239 per 100,000 live births [2, 3]. Despite the notable decrease, the MMR in Nepal is well above the global average of 216 and South East Asian average of 164 per 100,000 live births [4].

In Nepal, almost than two out of five maternal death and one out of two neonatal death occurs at home [3, 5]. Delivery is the most dangerous time for both the mother and her baby although the maternal death can occur at any point of time from pregnancy to 42 days after childbirth. In Nepal, the most common cause of maternal death is haemorrhage; the massive blood loss before reaching to the health facility [5].

The Ministry of Health and Population (MoHP) has been continuously expanding around the clock service delivery sites like birthing centres, Basic Emergency Obstetric and Neonatal Care (BEONC) and Comprehensive Emergency Obstetric and Neonatal Care (CEONC) sites to increase the access to emergency obstetric care and SBAs at birth [6]. The *Aama Surakshya* programme is in place to ensure free service, reduce demand side financial barriers and encourage women for institutional delivery. Apart from providing financial incentives to cover costs associated with institutional delivery and completing four antenatal care (ANC) visits to the service users, *Aama Surakshya* programme also provides incentives to health facilities for conducting deliveries and treating sick new-borns [6]. Nepal has to reduce MMR to 70 per 100,000 live births by the year 2030 to meet the Sustainable Development Goals (SDG) [7]. Realising the importance of universal access to skilled birth attendance as a crucial strategy to reduce maternal and neonatal mortality [8, 9], and reach the SDG goal of institutional birth to 90% by 2030; the Nepal Health Sector Strategy (NHSS) has the target of achieving institutional delivery of 70% by 2020. [10].

Evidence suggests an inequity in the utilisation of safe motherhood services including SBA delivery coverage, ANC, post-natal care (PNC) and new born care services. These indicators differ according to women’s socio-economic status, education level, place of residence, and geography and ethnicity [2, 10–13]. An equity oriented approach in achieving universal coverage stresses that an accelerated and early gains have to be realized by disadvantaged and marginalized population subgroups, thereby improving overall indicators of the country and reducing inequalities [14]. Moving from Millennium Development Goals (MDGs) to the SDGs, one of the major change in approach was the inclusion of the concept of leaving no-one behind [15]. Having understanding of whether the factors that lead to home delivery differ among marginalized and non-marginalized groups would provide opportunity to design targeted intervention in specific groups rather than having a blanket approach. For example, if the women empowerment or birth preparedness are found to be preventing women from having health facility delivery in marginalized group, policy makers may choose to design ethnic group specific innovative strategies. In this context, aimed to explore the determinants of home delivery among marginalised and non-marginalised women.

## Materials and methods

### Study setting

We used data from the 2016 Nepal Demographic and Health Survey (NDHS), which is a nationally representative community-based cross-sectional survey providing the most comprehensive data on basic demographic and health indicators. The survey was conducted from June 2016 to January 2017.

### Sampling and study population

In the original study, participants were selected using two stage cluster sampling in rural areas and three stage cluster sampling in urban areas (because of large size of urban wards). Wards are the smallest local administrative units that comprise a part of municipalities or rural municipalities. A total of 383 wards were selected using probability proportional to ward size with independent selection in each stratum. For urban wards, old wards (each new ward after administrative reform, is composed of few small old wards) were considered as enumeration area and one enumeration area was randomly selected. Household listing operation was carried out in the selected enumeration area, which was followed by selection of 30 households, using equal probability systematic selection. A detail methodology of NDHS has been presented elsewhere [2]. Altogether 11,040 households were enrolled in the survey. The study involved face to face interview using structured questionnaire as data collection technique.

Out of total 4,006 women of reproductive age (15 to 49 years) with at least one live birth in the five years preceding the survey, we excluded those women delivering in India (107 women), delivering on the way to health facility (52 women), and those who could not be classified as marginalised and non-marginalised based on information available (10 women). Thus, this article involves analysis of 2,418 woman in marginalised and 1,419 in non-marginalised group making total of 3,837 women of reproductive age group.

We categorised the women as marginalised or non-marginalised based on women’s ethnic group [16] reflective of the social hierarchy in Nepal as shown in Table 1. The Constitution of Nepal defines the excluded and vulnerable groups based on gender, age, caste, ethnicity, geography, mental and physical disability, economic status and facilitates to eliminate the discrimination against them and build an egalitarian society founded on proportional inclusive and participatory principles [17]. Ethnic categories included Dalits (Hill and Terai Dalits), Muslim, Janajati (Hill and Terai Janajati), Terai/Madhesi others, Brahmin/Chhetri (Hill and Terai Brahmin/Chhetri), Newar and others as shown in Table 1. Women from Dalit, Janajati (other than Newar), muslims and tarai caste group other than tarai bhramin and chhetri have been classified as marginalized.

**Table 1:** Categories defining marginalised and non- marginalised.

Category	Marginalised women	Non-marginalised women
1.	Terai Dalit	Hill Brahmin
2.	Hill Dalit	Hill Chhetri
3.	Hill Janajati	Terai Brahmin
4.	Terai Janajati	Terai Chhetri
5.	Muslim and Other Terai Caste	Newar
**Total number of women**	**2418 (63.02%)**	**1419 (36.98%)**
**Number (Percentage) of women delivering at home**	**1070 (46.78%)**	**450 (26.34%)**

### Study variables

Place of delivery is the dependent variable whereas independent variables include background variables, access related variables, women’s empowerment variables and pregnancy related variables as shown in Table 2.

**Table 2:** List of study variables and their operational definition.

Variables	Description of variables	Categories
Place of Delivery	Place of Delivery (dependent variable)	Home, Health Facility
**Background Variables**		
Age at last birth	Age in completed years of the women at the time of last child birth	15–19, 20–24, 25–29, 30 and above
Education	Level of education classified as per the years of schooling/grades completed	No education, basic education (those with incomplete secondary education and below), higher education (those completed secondary or higher)
Husband's education	Level of education classified as per the years of schooling/grades completed.	No education, basic education (those with incomplete secondary education and below), higher education (those completed secondary or higher)
Wealth quintile	Wealth quintile in five categories.	Poorest, poorer, middle, richer, and richest.
Place of residence		Urban and rural
Province	Geographical origin of the women	Province1, Province 2, Province 3, Province 4 (Gandaki Pradesh), Province 5, Province 6 (Karnali Pradesh) and Province 7 (Sudurpashchim Pradesh)
**Access related variables**		
Exposure to health programmes in media (heard seen at least 2)	Whether or not the women saw or heard at least two of the following health programmes: janasankhya chetana karyakaram, janaswasthya radio karyakram, jeevan chakra tv karyakram, saathi sanga mannka kura radio karyakram, thorai bhaye pugi sari tv karyakram, bhanchhin ama radio karyakram, parivaar niyojan smart banchha jeevan tv/radio karyakram, bhandai sundai radio karyakram, navi malam tv/radio karyakram	Yes, No
Distance to health facility	Duration to reach nearest health facility in minutes	>30 minutes and < = 30 minutes
Problem in health care access	Whether or not the women perceived the following as a problem: getting permission to go for treatment (medical help), getting money needed for treatment, distance to health facility, going alone to the health facility, concern of no female health provider	All five, some of them, none
**Women's empowerment**		
Women's empowerment	Whether or not women can refuse sex, women can decide on own healthcare, and can decide to use contraception	Yes, No
**Pregnancy related variables**		
Four ANC visits as per protocol	Whether or not women had received an ANC visit at 4, 6, 8 and 9 months among those with at least 1 ANC visit	Yes, No
ANC quality of care	Whether or not women were advised for SBA delivery, for institutional delivery, to look for possible problems with pregnancy, and to get post-natal check-up	Yes (all four), No (none or some)
Birth preparedness	Whether or not women were prepared with money, food, and clothes as a birth preparation.	Yes (all three), No (none or some)

### Statistical analysis

All analyses were performed using complex sample analysis considering the primary sampling unit, stratum and sample weight adjusting for selection probabilities. All statistical analyses were performed using STATA 15.0 for Windows. For statistical analysis, we ran bivariate logistic regression for each independent variable separately with the dependent variable before carrying out the multivariable analysis. Variables including exposure to media and timing of first ANC visit were excluded from the final multivariable model because of multi-collinearity. We present the results of multivariable logistic regression for marginalised women with adjusted odds ratio (AOR) and 95% confidence interval (CI).

### Ethical approval

This study is a secondary analysis of data from 2016 NDHS which is publicly available dataset from measure DHS website. NDHS had obtained ethical approval from Nepal Health Research Council (NHRC) and ethical review board ICF Macro International to conduct this study.

## Results

Among 3,837 women who delivered within the last five years, almost half of the marginalised women (47%) and one quarter of non-marginalised (26%) delivered their last child at home. Table 3 shows the proportion of women who had a home delivery by background characteristics. For both marginalised and non-marginalised women, the proportion of home delivery increased with increasing age and decreasing level of education, wealth quintile and husband’s education. The proportion of home delivery was largest among women aged 30–49 years (marginalised 59%; non-marginalised 30%). Among marginalised, the proportion of home delivery was least among Janajati (40%) with almost equal proportion (more than 50%) among Muslim, Dalit and other Terai ethnic groups whereas among non-marginalised, proportion of home delivery was lowest among Newar (24%) followed by Hill Brahmin/Chettri (26%) and Terai Brahmin/Chhetri (30%) ethnic groups. More than one in five non-marginalised women and one in three marginalised women below 20 years of age delivered their child at home. As compared to women with higher education (marginalised 20%; non-marginalised 10%), a larger proportion of women with no education (marginalised 63%; non-marginalised 62%) and basic education (marginalised 41%; non-marginalised 30%) delivered their last child at home. Home delivery was higher among women from rural areas (marginalised 57%; non-marginalised 45%), Province 2 (marginalised 58%; non-marginalised 33%) and Province 6 (marginalised 50%; non-marginalised 64%). Among marginalised women, home delivery rate was lowest (23%) in Province 7. The pattern of differences among provinces was different among non-marginalised women with the highest rate of home delivery in Province 6 (68%) and the lowest in Province 3 (11%).

**Table 3:** Percentage of marginalised and non- marginalised women delivering their last child at home, according to background variables.

Variables	Marginalised			Non-marginalised		
	n	%	95% CI	n	%	95% CI
**Age at Last Birth (years)**						
<20	549	34.35	29.28–39.80	227	22.18	16.42–29.24
20–24	918	47.54	43.00–52.12	588	25.11	20.42–30.47
25–29	574	49.03	44.08–54.00	382	28.18	23.14–33.85
30–49	377	58.74	51.91–65.25	222	30.15	22.26–39.43
**Caste/Ethnicity**						
Dalits	558	51.69	45.58–57.75	NA	NA	NA
Muslim	206	50.93	39.75–62.01	NA	NA	NA
Janajati	1138	39.85	38.83–45.09	NA	NA	NA
Other Terai	516	53.23	46.22–60.11	NA	NA	NA
Hill Brahmin/Chhetri	NA	NA	NA	1282	26.49	22.21–31.27
Newar	NA	NA	NA	101	24.41	14.97–37.21
Terai Brahmin/Chhetri	NA	NA	NA	36	29.67	12.70–55.01
**Education**						
No Education	877	63.45	58.70–67.96	293	62.44	52.82–71.17
Basic Education	1201	40.99	36.76–45.36	545	29.89	24.99–35.30
Higher Education	340	19.69	15.13–25.23	581	10.01	7.34–13.51
**Wealth Quintile**						
Poorest	501	66.41	58.97–73.11	481	59.83	51.07–68.00
Poorer	582	55.75	50.43–60.93	244	37.50	29.80–45.90
Middle	603	45.71	40.59–50.92	184	15.86	10.62–23.04
Richer	502	36.47	30.60–42.76	230	10.86	6.93–16.64
Richest	230	16.67	11.36–23.79	280	2.862	1.31–6.12
**Husband's Education**						
No Formal Education	402	65.56	59.78–70.91	72	74.75	58.67–86.06
Basic Education	1447	46.77	42.75–50.82	636	34.44	28.87–40.48
Higher Education	549	31.30	26.49–36.54	696	15.41	12.22–19.26
**Place of Residence**						
Rural	1028	57.31	51.98–62.48	559	45.09	12.01–20.21
Urban	1390	37.06	32.14–42.27	860	15.68	37.50–52.92
**Province**						
Province 1	364	40.43	33.65–47.59	185	23.16	14.06–35.70
Province 2	670	57.83	51.35–64.06	39	32.64	14.72–57.63
Province 3	241	42.60	31.05–54.92	182	10.9	6.14–18.61
Province 4	280	39.86	29.09–51.71	149	12.95	7.60–21.23
Province 5	449	44.19	35.86–52.85	170	20.04	12.91–29.76
Province 6	213	50.24	36.64–63.80	374	64.1	53.47–73.50
Province 7	201	22.97	16.11–31.66	320	36.69	26.32–48.47
** Ecological zone**						
Mountain	83	50.49	31.16–69.67	237	56.69	41.84–70.43
HIll	855	45.54	39.43–51.79	830	25.99	20.98–31.72
Terai	1480	47.15	42.76–51.59	352	11.69	8.19–16.42
** Total**		**46.78**			**26.34**	

Home deliveries were observed in greater proportions among marginalised compared with non-marginalised women. Home deliveries were more common in marginalised women with basic education (50% compared to 30% of non-marginalised women); with higher degree of education (20% compared to 10% of non-marginalised), richer wealth quintile (36% compared to 11% non-marginalised) or richest wealth quintile (17% compared to 3% non-marginalised) and with urban residence (37% compared to 16% non-marginalised).

Table 4 presents the proportion of women who delivered their last child at home by access-related variables and women’s empowerment. In both groups of women, the proportion of home delivery was higher among the women who did not have exposure to health programmes (marginalised 52%; non-marginalised 38%). Nonetheless, it was also common among a significant proportion of women (marginalised 33%; non-marginalised 19%) having exposure to health programmes. The proportion of home delivery was greater among women who lived more than 30 minutes walking distance to health facility (marginalised 56%; non-marginalised 42%), however it is interesting to note even among the women with less than 30 minutes walking distance to the health facility, home delivery was 43% among marginalised women and 18% among non-marginalised women. Similarly, the home delivery rate was higher among women who felt problems related to health care access and those who were not empowered in both marginalised and non-marginalised categories.

**Table 4:** Percentage of marginalised and non- marginalised women delivering their last child at home, according to access to health care and women empowerment.

Variables	Marginalised			Non-marginalised		
	n	%	95% CI	n	%	95% CI
**Exposure to Health Programmes**						
No	1657	52.26	48.35–56.15	585	38.38	31.71–45.52
Yes	761	33.17	28.52–38.17	834	18.92	15.39–23.04
**Distance to HF**						
30 min or less	1676	43.04	39.17–47.01	829	17.88	14.10–22.42
More than 30 min	739	56.23	49.98–62.28	589	42.09	35.11–49.40
**Problem in Health Care Access**						
All 5 Problems	596	53.42	28.03–42.38	234	43.93	33.86–54.52
Some	1528	46.31	42.07–50.60	912	27.44	23.04–32.32
None	294	34.87	47.91–58.84	273	11.56	7.47–17.48
**Women's Empowerment**						
No	1330	49.14	44.99–53.31	721	31.56	26.36–37.27
Yes	914	42.16	37.74–46.71	613	20.13	22.00–30.23
** Total**		**46.78**			**26.34**	

Nevertheless, the proportion of home delivery was similar among women with problems in healthcare access and among women who reported that they were not empowered.

Table 5 shows the proportion of women who delivered their last child at home by completion status of ANC care and whether they had birth preparedness. The rate of home delivery was higher among the women who had not completed four ANC visits (marginalised 58%; non-marginalised 46%) compared to those who completed (marginalised 31%; non-marginalised 16%). A larger proportion of women who did not receive quality care during ANC (marginalised 50%; non-marginalised 31%) and with no birth preparedness (marginalised 50%; non-marginalised 31%) delivered at home.

**Table 5:** Percentage of marginalised and non- marginalised women delivering their last child at home, according to ANC and birth preparedness variables.

Variables	Marginalised			Non -marginalised		
	n	%	95% CI	n	%	95% CI
**4 ANC Visits as Per Protocol**						
Not completed	1018	58.30	53.93–62.54	409	45.74	38.87–52.79
Completed	1239	31.09	27.64–34.76	934	16.41	13.13–20.34
**ANC Quality of Care**						
No	1215	49.84	45.36–54.33	508	31.05	25.31–37.41
Yes	1032	36.70	32.66–40.93	827	20.13	16.31–24.58
**Birth Preparedness**						
No	1746	50.17	46.15–54.18	865	31.31	26.17–36.95
Yes	672	37.58	32.46–43.00	554	18.73	14.50–23.85
** Total**		**46.78**			**26.34**	

Women who delivered their most recent child at home were asked for the reasons for not delivering in the health facility. Table 6 shows that the most commonly reported reason was that it was not necessary to deliver in a health facility. This was followed by the birth taking place before reaching the facility, the facility being too far away or not having transportation. As compared to non-marginalised women (35%), a large proportion of marginalised women (64%) felt that it was not necessary to give birth at health facility. Whereas all other reasons were more commonly cited by a greater proportion of non-marginalised as compared to marginalised women. It is notable that among both marginalised and non-marginalised women, only 2% of women reported cost as the reason for not delivering in the health facility. Issues relating to the quality of health services such as ‘facility not open’, ‘do not trust/poor service’, or ‘no female provider’ were also raised by non-marginalised women than marginalised women.

**Table 6:** Reasons for not delivering in the health facility among marginalised and non-marginalised women who delivered their last child at home.

Variables	Marginalised (n = 1070)		Non -marginalised (n = 450)	
	%	95% CI	%	95% CI
Not necessary	64.16	59.7–68.4	35.47	29.2–42.2
Child born before reaching facility	13.73	11.2–16.7	22.65	18.0–28.1
Too far/no transport	12.62	9.9–16.0	33.46	26.5–41.2
Not customary	6.30	4.7–8.4	11.83	7.4–18.3
Husband/family did not allow	3.63	2.5–5.3	1.07	0.2–4.6
Cost too much	1.99	1.2–3.2	2.17	0.9–4.9
Facility not open	1.45	0.9–2.5	2.16	0.9–4.9
Do not trust/poor service	0.82	0.4–1.6	2.82	1.1–7.1
No female provider	0.68	0.3–1.4	1.32	0.5–3.2
Other	4.31	3.1–5.9	6.59	3.9–11.1

Table 7 presents the result on bivariate and multivariable logistic regression, which illustrates the odds of delivering at home for the last-born child among marginalised and non-marginalised. The multivariable analysis indicated an independent association of maternal age at birth, education, wealth quintile and completion of ANC protocol with home delivery among both marginalised and non-marginalised women. Place of residence and the distance to health facility were also independently associated with home delivery among marginalised women.

**Table 7:** Bivariate and multivariable analysis using logistic regression for assessing the factors associated with home delivery among marginalised and non-marginalised women.

Variable	Non marginalized				Marginalized			
Categories	Unadjusted OR	95% CI	Adjusted OR	95% CI	Unadjusted OR	95% CI	Adjusted OR	95% CI
**Age at Last Birth**								
<20	Referent		Referent		Referent		Referent	
20–24	1.18	0.78–1.79	1.65	1.01–2.7	1.73	1.34–2.24	2.03	1.47–2.81
25–29	1.38	0.93–2.03	1.9	1.1–3.28	1.84	1.4–2.42	2.13	1.47–3.08
30–49	1.52	0.98–2.34	0.86	0.47–1.57	2.72	1.93–3.85	2.46	1.6–3.79
**Education of participant**								
Higher education	Referent		Referent		Referent		Referent	
No Education	14.94	9.34–23.9	4.13	2.07–8.22	7.08	4.96–10.1	2.27	1.45–3.55
Basic Education	3.83	2.6–5.65	2.18	1.39–3.43	2.83	2.01–4	1.59	1.06–2.39
**Husband's Education**								
Higher education	Referent		Referent		Referent		Referent	
No Education	16.25	7.45–35.44	2.31	0.88–6.05	4.18	3.03–5.76	1.41	0.95–2.08
Basic Education	2.88	2.22–3.75	0.93	0.65–1.33	1.93	1.54–2.42	1.28	0.96–1.69
**Place of residence**								
Urban	Referent		Referent		Referent		Referent	
Rural	4.42	2.83–6.9	1.41	0.86–2.3	2.28	1.67–3.11	1.91	1.4–2.6
**Wealth Quintile**								
Richest	Referent		Referent		Referent		Referent	
Poorest	50.55	21.15–120.79	11.47	3.62–36.29	9.88	5.74–17.01	5.46	2.87–10.39
Poorer	20.37	8.33–49.79	10.9	3.8–31.25	6.3	3.83–10.35	3.4	1.99–5.81
Middle	6.4	2.57–15.96	3.44	1.2–9.83	4.21	2.6–6.82	1.85	1.13–3.06
Richer	4.14	1.64–10.45	2.79	0.99–7.92	2.87	1.77–4.65	1.59	0.95–2.66
**Province**								
Province 3	referent		Referent		Referent		Referent	
Province 1	2.46	1.03–5.91	1.75	0.72–4.25	0.92	0.52–1.63	0.97	0.52–1.78
Province 2	3.96	1.19–13.24	5.33	1.41–20.15	1.85	1.06–3.25	2.16	1.08–4.31
Province 4	1.22	0.51–2.88	0.95	0.37–2.46	0.9	0.45–1.79	0.86	0.46–1.63
Province 5	2.05	0.91–4.64	1.31	0.53–3.23	1.07	0.58–1.96	1.41	0.72–2.79
Province 6	14.59	6.79–31.36	2.64	1.16–6.02	1.36	0.65–2.88	0.76	0.35–1.64
Province 7	4.74	2.15–10.45	1.2	0.48–2.99	0.4	0.21–0.78	0.39	0.18–0.87
**Exposure to Health Programmes**								
Yes	Referent		Referent		Referent		Referent	
No	2.67	1.88–3.79	0.99	0.68–1.46	2.21	1.77–2.75	1.17	0.87–1.59
**Distance to HF**								
30 min or less	Referent		Referent		Referent		Referent	
More than 30 min	3.34	2.26–4.93	0.97	0.66–1.42	1.7	1.28–2.25	1.35	1–1.82
**Problem in Health Care Access**								
None	Referent		Referent		Referent		Referent	
Some	2.89	1.72–4.87	0.96	0.53–1.73	1.61	1.14–2.27	0.93	0.64–1.36
All 5 Problems	5.99	3.06–11.72	0.74	0.34–1.62	2.14	1.49–3.09	0.94	0.61–1.46
**4 ANC Visits as per Protocol**								
Completed	Referent		Referent		Referent		Referent	
Not completed	4.29	3.07–6.01	2.33	1.61–3.36	3.1	2.52–3.81	2.25	1.77–2.86
**ANC Quality of Care**								
Yes	Referent		Referent		Referent		Referent	
No	1.79	1.28–2.5	1.32	0.9–1.92	1.71	1.38–2.13	1.09	0.85–1.39
**Birth Preparedness**								
Yes	Referent		Referent		Referent		Referent	
No	1.98	1.41–2.78	1.45	0.93–2.26	1.67	1.31–2.14	1.23	0.95–1.59
**Women's Empowerment**								
Yes	Referent		Referent		Referent		Referent	
No	1.83	1.29–2.6	1.17	0.8–1.72	1.33	1.09–1.62	1.14	0.9–1.45
**Ecological zone**								
Terai	Referent		Referent		Referent		Referent	
Mountain	0.27	0.14–0.53	1.83	0.74–4.54	1.14	0.50–2.63	1.46	0.64–3.36
Hill	0.1	0.05–0.21	1.76	0.92–3.35)	0.93	0.69–1.27	1.22	0.74–2.02

Among marginalised women, we found a significant association between mothers’ age and home delivery. As compared to women under 20 years of age, women with age 20–24 years [AOR: 2.03 (95% CI: 1.47–2.81)], 25–29 years [AOR: 2.13 (95% CI: 1.47–3.08)] and 30–49 years [AOR: 2.46 (95% CI:1.60–3.79)] had higher odds of delivering at home. The women who had no education had 2.27 times (95% CI: 1.45–3.55) and with basic education had 1.59 times (95% CI: 1.06–2.39) higher odds of having home delivery—than those who had higher education. We found that, compared to women from the richest wealth quintile, the odds of having a home delivery was 5.46 times (95% CI: 2.87–10.39) higher among those belonging to poorest wealth quintile, 3.40 times (95% CI: 1.99–5.81) higher among women from poorer wealth quintile and 1.85 times (95% CI: 1.13–3.06) higher among women in middle wealth quintile. The odds of home delivery were 1.91 times (95% CI: 1.40–2.60) more among women residing in rural areas in comparison to those residing in urban areas. As compared to Province 3, province 2 had 2 times higher odds (95%CI: 1.08–4.31) and province 7 had lower odds [AOR 0.39 (95%CI: 0.18–0.87)] of delivering at home. Women residing at greater walking distance (>30 minutes) to nearest health facility [AOR: 1.35 (95% CI: 1.00–1.82)] had higher odds of having home delivery than those residing at distance equals to or less than 30 minutes. Women who did not completed ANC visit as per protocol had greater odds of delivering at home [AOR:2.25 (95% CI: 1.77–2.86)].

Similarly, among non-marginalised women, the effect of mothers’ age at birth were visible only after multivariable analysis. As compared to women below 20 years of age, women of age 20–24 years had 1.65 times (95% CI: 1.01–2.70) and 25–29 years had 1.90 times (95% CI: 1.10–3.28) higher odds of having home delivery. The odds of home delivery were four times higher (95% CI: 2.07–8.22) among women with no education and two times higher (95% CI: 1.39–3.43) among women with basic education. As compared to women from richest wealth quintile, the odds of having home delivery was 11.47 times higher (95% CI: 3.62–36.29) among women from poorest quintile, 10.90 times higher (95% CI: 3.80–31.25) among women from poorer wealth quintile, 3.44 times higher (95% CI: 1.20–9.83) among women from middle wealth quintile. Women residing in Province 6 (Karnali Pradesh) had 2.64 times (95% CI: 1.16–6.02) higher odds and Province 2 had 5.33 times (95% CI: 1.41–20.15) higher odds of having home delivery as compared to Province 3. Women who did not complete four ANC visit as per protocol had 2.33 times higher odds (95% CI: 1.61–3.36) of giving birth to baby at home. Factors associated with home delivery were similar among marginalized poor, marginalized poor and illiterate women. Detail findings have been presented in S1 Appendix

## Discussion

Delivery care services are major components of the Safe Motherhood Programme. We observed that nearly half of the marginalised women (47%) and slightly more than one quarter of non-marginalised women (26%) delivered at home. The proportion for marginalised women is slightly higher than the national average of 41% [2].

Evidence suggests that maternal and neonatal mortality is associated with the complication that may arise any time during child birth without SBA [18–21]. In some of the developed countries, there is increasing trend of planned home delivery with evidence suggesting no difference in neonatal mortality and morbidity between planned home deliveries and hospital births [22–24]. However, in resource poor settings like Nepal where SBA are able to reach only 6.5% of deliveries outside health facilities, strategies to ensure presence of SBA to assist deliveries at home can be less feasible [3]; i.e., home delivery in Nepal means that most of the mothers are giving birth to their child without any skill attendant or with an untrained friend or relative. Among South Asian countries, the proportion of births without any skilled health personnel in Nepal (42%) is more than India (12%), Bhutan (11%), Maldives (4%) and Sri Lanka (1%) and less than Afghanistan (49%), Bangladesh (50%) and Pakistan (48%) [25]. This highlights that there is lot of space for improvement in maternal health through promotion of institutional delivery in Nepal compared to other countries in South Asia.

This is the first study reporting factors associated with home delivery disaggregated by ethnicity. Our study also shows higher proportion of home delivery among marginalised (40% among Janajati, more than 50% among each of Muslim, Dalit and other Terai caste group) as compared to non-marginalised (24% among Newar, 26% among Hill Brahmin/Chettri and 30% among Terai Brahmin/Chhettri) women. The ethnicity reflects the important socio-cultural structure of Nepalese society. A systematic review on ethnicity and maternal health outcomes and service coverage in China shows that women from ethnic minorities were less likely to utilise maternal and child care services [26]. Similarly, studies from India and Tanzania suggest that scheduled castes were more likely to have home delivery as compared to general castes [27–29]. In one of the studies conducted in Chitwan district of Nepal, the odds of delivering a child at a health facility was two times higher among advantaged ethnic groups compared to disadvantaged ethnic groups [30]. These disparities have been persisting for more than a decade [11, 16].

In this study, there is an independent association of lower education level, poorer wealth quintile and non-completion of four ANC visits as per national protocol with home delivery among both marginalised and non-marginalised women. Place of residence, and the distance to health facility—which are to some extent variables related to geography—were independently associated with home delivery only among marginalised women. We could not find any previous studies comparing factors associated with home delivery disaggregated by ethnicity in Nepal. However, several studies demonstrated statistically significant associations of home delivery with mothers age at birth [31], educational level of the mother [32–34], wealth status [31, 33], province [33], place of residence [31, 33], travel time to nearest health facility [32, 34, 35], and completion of four ANC visits as per national protocol [31, 32, 34, 36].

Among both marginalized and non-marginalized, women of age 20–24 years and 25–29 years had higher odds of delivering at home. This might be because of the birth order of women/ multiparity, as multiparous women tend to have precipitation of labour [32, 37]. Further, previous literature shows multiparity being strongly associated with unplanned birth and the relationship between age and unplanned birth is confounded by parity [38]. Women in upper age group or with multiparity should be encouraged to have institutional delivery. However, while doing so, the teenagers, who often tend to have high-risk pregnancies also should not be left behind.

Our analysis showed poor educational status as one of the risk factors for home delivery among both marginalised and non-marginalised women. The women who had no education and basic education had greater odds of having a home delivery than those who had higher education. Other studies too have shown that institutional delivery is higher among educated people and those with better income status than the uneducated and poor [39–41]. This might be because educated women comprehend better about the potential risk associated with home delivery and have better idea about the service availability. The women with low education could be made aware of the risks related to home delivery without SBA, which might increase the health facility delivery. However, since previous literature shows the prominent role of mother-in-law and husband in decision making of seeking maternal health care services, it would be important to educate them too [42, 43].

In both groups, economic status was also an important predictor of home delivery. Poverty is one of the key factors associated with low utilisation of health care services in Nepal and in other developing countries [39, 40]. But very few women, both from marginalised and non-marginalised (close to 2%) reported cost as the reason for not delivering in health facility. Economic status might have acted through some other intermediate variables not have been considered in the study. In Nepal, *Aama Surakshya* Programme is providing transportation incentive for women delivering in the health facility to overcome barriers relating to cost. In this context, to have better understanding of how economic status plays role in choice of place of delivery for giving birth, further studies can be useful.

Among marginalised women, with reference to province 3, province 2 had higher odds while province 7 had lower odds of delivering at home. Province 2 includes 8 Terai districts from Southern Nepal which are largely inhabited by the marginalised ethnic population—“Madheshi” [44]. Previous studies showed that access to family planning services, ANC, and use of SBAs during deliveries was lower among the Terai dalit and janajati in comparison to other ethnic groups [11, 16]. Women in Province 2 might have faced problems in accessing health services despite better availability because of being socially, culturally and economically excluded from mainstream development [45]. Among non-marginalised, compared to the women residing in Province 3, the women from Province 6 had almost three times higher odds of having home delivery. Province 6 is the most underdeveloped region of the country and has the highest multidimensional poverty index [46] and including poor health indicators for nutrition, ANC coverage, neonatal mortality, family planning etc. The public health facilities are also at farther distance from the households in comparison to other provinces [2]. There are studies from within the country and from across the globe which show that some sociocultural barriers are associated with utilisation of maternity service [43, 47, 48]. This study involved secondary analysis of the 2016 NDHS and thus could not assess all the factors that might have contributed to home delivery. Future research exploring a wider range of socio-demographic variables would be appropriate.

Failing to complete at least four ANC visits as per national protocol was found to be associated with home delivery among both groups. The women who had not completed four ANC visits had more than two times higher odds of having home delivery compared to those who completed. This emphasises the importance of getting women in contact of the health services during pregnancy which could ultimately also result in women returning to the health facility for giving birth. Several studies demonstrate the positive effects of antenatal care on institutional delivery [31, 32, 34, 36].

However, the descriptive finding shows that 16% of non-marginalised women, who completed four ANC visits had home delivery. Having a home delivery despite completing ANC visits can also be related to satisfaction of patients to ANC services. A qualitative study from Ethiopia indicates poor counselling during antenatal care as the cause of home delivery among women with ANC [49]. Further research in the Nepalese context can be useful to identify why women deliver at home despite having ANC visits. Similarly, it was observed that a greater proportion of women were delivering at home among marginalised as compared to non-marginalised even when they were educated, belonged to richer wealth quintile, lived in urban areas, which might suggest that there are some other factors responsible for marginalised women for having home delivery.

Rural residence was associated with higher home delivery rates only among marginalised women. Factors like cultural and religious beliefs might have been stronger in rural areas among marginalised groups. This finding is consistent with other studies [31, 33].

Likewise, in this study, marginalised women located with their health facility at farther distance (>30 minutes) had higher odds of delivering at home, whereas it was not significant among non-marginalised women. Distance to health facility was also found to have significant effect for place of delivery in previous studies [32, 34, 35].

Descriptive findings from this study also shows that not feeling the necessity of delivering in health facilities, birth taking place before reaching health facility and distance to health facility were the top three reasons for not delivering in a health facility, which is congruent with other DHS studies [50]. The reason of not feeling necessary to deliver in health facilities contributed the highest proportion among both marginalised (64%) and non-marginalised women (35%). This might be because of lack of awareness and understanding among women regarding the consequences of delivering their child at home without a skilled attendant and the benefits of institutional delivery, or it might be because of socio-demographic or cultural factors as illustrated by the study from Nigeria and Ethiopia which suggested lower likelihood of considering facility delivery as unnecessary among women with higher educational level [51]. It might be partly a reflection of their perception of the quality of services being delivered in health facilities as there is notable proportion of women who visited health facilities for ANC services but delivered babies at home. A possible strategy to be adopted by MoHP could be to increase the awareness level especially with a greater focus on marginalised women and improve the quality of services being delivered in health facilities.

Nepal has made significant progress in improving maternal and child health as evidenced by the increase in institutional birth rate and decreased MMR and neonatal mortality rate. The institutional delivery rate in Nepal is 58% and thus the SDG goal to reach institutional birth to 90% by 2030 presents a major challenge. The health system of Nepal can expedite its performance in increasing institutional delivery among both group of women, if it could address the factors associated with home delivery as illustrated in this study- encouraging four ANC visits and prioritising initiatives that target women’s empowerment that are sensitive to ethnic diversity. Based on the findings of this study, we can infer that to increase maternal health care access to marginalised women, then women residing in the rural areas, residing at Province 2 and 5 and at distant location from health facilities should be targeted in strategies to promote maternal health. The ‘National Strategy on Reaching the Unreached’, outlines practical ways to improve access and use of quality health services to marginalised populations across different ethnic groups [52]. The present context of federalism where service delivery has been devolved to health facilities at provincial and local level can be an opportunity to design a strategy that reflects the local context. When comparing with other countries, the Democratic Republic of Congo has 91% deliveries by SBA, but MMR is 442 per 100,000 live births; Djibouti has more than 87% of deliveries by SBA but the MMR is same as that of Nepal [53]; which indicates that that increasing the rate of institutional delivery alone might not decrease the maternal death. This hints to the issues of supply side barriers or quality of care in these countries. Therefore, quality of care, which this study could not assess in detail, should also be taken into account during the efforts to increase institutional delivery. Acknowledging the importance of quality, the Government of Nepal/Ministry of health and population, in order to improve maternal and neonatal health status, has been working on Safe motherhood and neonatal health (SMNH) roadmap which currently is in the draft stage and considers quality as central principle. There might be some bias associated with dichotomization of variables like ANC quality of care and birth preparedness. Furthermore, apart from technical quality, perceived quality of service could be important in determining which has not been covered by the study.

## Conclusion

A larger proportion of marginalised women as compared to non-marginalised give birth at home. Marginalised and non-marginalised women have similar determinants (lower education status, poorer economic status, non-completion of ANC visits as per protocol) for home delivery/ challenges for institutional delivery, however the geographical barriers (rural residence, belonging to Province 7, and residing at a distance of >30 minutes to health facility) were pertinent only among marginalised women. Since there are more marginalised women delivering at home, this group should be targeted for interventions. Considering a substantial proportion of women delivering at home, improved awareness through tailored behaviour change communications, including targeting the mother-in-laws and husbands to have better understanding of the risk associated with unattended home delivery could help women shifting from home to health facility for delivery.

There should be further studies to explore other factors–such as quality of services (including antenatal care services), sociocultural factors and other motivators for institutional delivery -which might have important contribution in home delivery.

## Supporting information

S1 Appendix Analysis of determinants of home delivery among marginalized and non marginalized women based on ethnicity, wealth quintile and literacy status. (XLSX)

S1 Data Caste and ethnic group, Nepal—Source: Bennett L, Dahal DR, Govindasamy P. 2008(12). (DOCX)
