# Supplementary material for: Determinants of home delivery in Nepal – A disaggregated analysis of marginalised and non-marginalised women from the 2016 Nepal Demographic and Health Survey
Source: PLoS One. 2020 Jan 30;15(1):e0228440. doi: 10.1371/journal.pone.0228440 (PMC6992204; doi:10.1371/journal.pone.0228440)
Supplement: S1 Data — (DOCX) [file pone.0228440.s002.docx]

## Supporting information

Caste and Ethnic Group, Nepal - Source: Bennett L, Dahal DR, Govindasamy P. 2008(12)

| Main Caste/Ethnic Groups (7) | Caste/Ethnic Groups with Regional Divisions (11) and Social Groups (103), 2001 Census |
| --- | --- |
| 1. Brahmin/Chhetri | - 1. Hill Brahmin |
|  | - 1. Hill Chettri   Chettri, Thakuri, Sanyasi |
|  | - 1. Terai/Madhesi Brahmin/Chhetri   Madhesi Brahmin, Nurang, Rajput, Kayastha |
| 1. Terai/Madhesi Other | 1. Terai/Madhesi Other   Kewat, Mallah, Lohar, Nuniya, Kahar, Lodha, Rajbhar, Bing, Mali Kamar, Dhuniya, Yadav, Teli, Koiri, Kurmi, Sonar, Baiya, Kalwar, Thakur/Hazam, Kanu, Sudhi, Kumhar, Haluwai, Badhai, Barai, Bhediya/Gaderi |
| 1. Dalits | - 1. Hill Dalit   Kami, damai/Dholi, sarki, badi,gaine, unidentified dalits |
|  | - 1. Terai/Madhesi Dalit   Chamar, Harijan, Musahar, Durshad/Paswan, Tatma, Khatwe, Dhobi, Baantar, Chidimar, Dom, Halkhor |
| 1. Newar | 1. Newar |
| 1. Janajati | - 1. Hill/Mountain Janajati   Tamang,Kumar, Sunuwar, Majhi, Danuwar, Thami/Thangmi, Darai, Bhote, Baramu/Bramhu, Pahari, Kusunda, Faji, Raute, Chepang/Praja, Hayu, Magar, Chyantal, Rai, Sherpa, Bhujel/Gharti, Yakha, Thakali, Limbu, Lepcha, Bhote, Byansi, Jirel, Hyalmo, Walung, Gurung, Dura |
|  | - 1. Terai Janajati   Tharu, Jhangad, Dhanuk, Rajbanshi, Gangai, Sathal/Satar, Dhimal, Tajpuriya, Meche, Koche, Kisan, Munda, Kusbadiya/Patharkata, Unidentified Adibas/Janajati |
| 1. Muslim | 1. Muslim   Madhesi Muslim, Churoute (Hill Muslim) |
| 1. Other | 1. Other   Marwari, Bangali, Jain, Punjabi/Sikh, Unidentified Others |
